# Patient engagement as a collaborative process in a large Dutch COVID-19 vaccination study (RECOVAC) – insight into the contribution of patient engagement and learnings for the future

**DOI:** 10.1186/s40900-024-00622-x

**Published:** 2024-09-13

**Authors:** J. P. M. Vervoort, W. S. Konijn, D. E. M. C. Jansen, C. Boersma, J. de Zeeuw, M. M. Ho-dac – Pannekeet, R. T. Gansevoort, A. L. Messchendorp, J. S. F. Sanders, R. de Wildt-Liesveld

**Affiliations:** 1 Dutch Kidney Patients Association (NVN), Bussum, The Netherlands; 2 Department of Health Sciences, University Medical Center Groningen, University of Groningen, Groningen, The Netherlands; 3 Department of Primary Care and Long Term Care, University Medical Center Groningen, University of Groningen, Groningen, The Netherlands; 4 Department of Management Sciences, Open University, Heerlen, The Netherlands; 5 Department of Internal Medicine, Division of Nephrology, University Medical Center Groningen, University of Groningen, Groningen, The Netherlands

**Keywords:** Patient engagement, Patient engagement monitoring and evaluation (PEME) framework, COVID-19 vaccination, Kidney patients, Collaborative process

## Abstract

**Background:**

The need for patient engagement in health research has been increasingly acknowledged and accepted in recent years. However, implementation is still limited due to lack of evidence on its value and lack of guidance on how to implement patient engagement. This study aims to provide insight into the contribution of patient engagement in the RECOVAC project, which studied COVID-19 vaccination in kidney patients, and formulate concrete practice-based action perspectives for patient engagement.

**Methods:**

We used a qualitative participatory mixed methods approach, based on the Patient Engagement Monitoring and Evaluation (PEME) framework. Patient engagement and data collection were based on the Reflexive Monitoring in Action (RMA) approach. Data collection included participant observations, open ended questionnaires and interactive reflection sessions. Qualitative analysis was done via a thematic approach.

**Results:**

We have described the process of patient engagement systematically, provided insight in its value and found that there is a need for clear aims, expectations and preparations from the start of the engagement process. We have shown that reflection throughout the process is of utmost importance and the same applies to clear communication between researchers and patient representatives. By being part of the consortium patient representatives had direct access to information, straight from the source, on for example the vaccination schedule and medication availability and had indirect influence on decisions made by the National Institute for Public Health and the Environment (RIVM) on preventive measures and treatment against COVID-19. Having experienced patient representatives is important, otherwise training needs to be provided. We also found that patient engagement had impact on conduct and outcomes of research activities itself and may have impact on future research and patient engagement activities in general.

**Conclusion:**

Patient engagement has changed the course of the project. Concrete practice-based action perspectives have been formulated, which are already being implemented by the Dutch Kidney Patients Association (NVN). Studying patient engagement in a high pace project with high public interest has resulted in lessons learned and will help prepare and implement patient involvement in future research projects.

**Clinical trial registration:**

The RECOVAC studies in which the patient engagement took place are registered at clinicialtrial.gov (NCT04741386 registration date 2021-02-04, NCT04841785 registration date 2021-03-22 and NCT05030974 registration date 2021-08-20).

**Supplementary Information:**

The online version contains supplementary material available at 10.1186/s40900-024-00622-x.

## Abstract (plain language summary)

This article is about the extensive engagement of patients in a scientific research project and what that engagement adds to the project. Although researchers acknowledge the importance of engagement of patients in research projects, it is not happening very often, Because there is not enough scientific evidence on the value of patient engagement and not enough guidance for researcher on how to implement it in their research. We used the Patient Engagement Monitoring and Evaluation (PEME) framework and qualitative participatory mixed methods research to provide insight into patient involvement in the RECOVAC project, which studied COVID-19 vaccination in kidney patients. We also formulated practical guidance for researchers who want to engage patients in their research. We describe the process of patient engagement in the RECOVAC project; what went well and what could be improved. We found that it is important to prepare well, keep reflecting on the engagement process throughout the project with all stakeholders of the project, communicate clearly and have experienced patient representatives involved or have training available for them. Patient engagement had impact on the conduct and outcome of the research activities itself and on activities outside of the project (e.g., doctors changing their conversations with their patients). We can conclude that involving patients changed the project and its outcomes to better fit with the needs of patients. A guideline has been made and is already implemented by the Dutch Kidney Patients Association. The lessons learned from this project will help researchers involve patients in their future projects.

**Supplementary Information:**

The online version contains supplementary material available at 10.1186/s40900-024-00622-x.

## Background

Over the past decades, integrating patient perspectives and insights into health research has been increasingly acknowledged and accepted [1]. This is referred to as patient engagement [2, 3] and is based on two types of arguments. First, on the normative argument that patients have the right to be involved in health research that affects them directly. This argument builds on the ethical principle often brought in by patients: ‘*nothing about us*,* without us’* [4]. The second argument is of a more political nature and recognizes that patient engagement in health research can be expected to enhance the quality and applicability of the research outcomes in healthcare practice [5–8].

Although the importance of patient engagement in health research is increasingly acknowledged and accepted, its actual implementation is still limited [2, 9–11]. One major reason is that embedding and systematically implementing patient engagement in research is often perceived as challenging. This may be partly due to limited evidence as well as the lack of consensus on the value of patient engagement [12–14]. To address this need for more evidence, an increasing number of studies aims to formulate evaluation frameworks [15–17]. An example of such a framework is the Patient Engagement Monitoring and Evaluation framework (PEME) [12]. This relatively new framework has been created, together with different stakeholders, to help patient engagement initiatives within medicine development to evaluate the progress and impact of patient engagement.

A second reason that contributes to the relatively slow implementation of patient engagement in health research is that researchers, despite the numerous frameworks to guide the set-up and execution of patient engagement, experience challenges in the implementation of these frameworks [15, 18]. Much of the existing guidance is too generic making it difficult for researchers to apply these general recommendations to their specific context [18, 19]. Researchers may find it difficult to formulate what approaches work well in which contexts, whom to engage, where to find patient representatives, and how to collect and integrate their perspectives in a valuable and sustainable way [7, 9, 20]. This gap between the availability of general recommendations and the need for concrete action perspectives may result in high ‘functional task uncertainty’ [21], in which the links between activities and the purpose and impact of patient engagement are unclear. To reduce the level of functional task uncertainty, there is a need for practice based ‘how to’ guidance on patient engagement based on the implementation of the general recommendations in specific contexts. Although some “how to” guidance has already been developed [22–24], more guidance based on real life case studies of patient engagement will increase the strength of evidence for its incorporation in health research.

To contribute to the systematic implementation of patient engagement in research this study aims to (1) provide insight into the contribution of patient engagement in a specific health research project using the PEME framework and (2) formulate concrete, practice-based action perspectives for patient engagement in health research, both for researchers and engaged patients.

## Methods

This study aims to provide insight into the contribution of patient engagement in the specific context of the RECOVAC (Renal Patients COVID-19 VACcination) consortium [25] and formulate concrete, practice-based action perspectives based on the outcomes. The RECOVAC consortium was established to evaluate and improve the immunogenicity of COVID-19 vaccination in kidney patients with either severely impaired kidney function, on dialysis or living with a kidney transplant. The consortium aimed to perform several observational and interventional studies [26–31].

### Description of the consortium

The RECOVAC-consortium is a collaboration between all seven university medical centers in The Netherlands. Collaborators within the consortium are clinical and non-clinical researchers at different stages of their career (e.g., PhD students, postdocs, professors) from the departments of Nephrology, Microbiology and Immunology. The core team of the consortium comprises the principal clinical and immunological investigators of each center, their PhD students and the project coordinators. The project coordinators and their team had most experience with involving patients in research and were therefore responsible for the implementation of patient engagement. The other researchers, PhD students and lab technicians had a more executive role within the patient engagement process without direct responsibilities for management and implementation.

Four patient representatives participated as full members of the consortium from the start of the project (Q1 2021) and one patient representative joined later (Q4 2021). The five patient representatives were recruited based on the following criteria: different medical history (e.g., dialysis, transplantation, being a relative), diversity in the complexity of their medical history, diversity in the age of onset of the disease, diversity in age and/or gender. Also, all patient representatives had multiple years of experience with patient engagement and were able to actively participate in a high paced project and bridge the gap between research and daily practice. They were supported by two policy officers of the Dutch Kidney Patients Association (NVN). The patient representatives participated in all stages of the research projects after funding was granted, as they were full members of the consortium and patient engagement was intertwined in all activities.

### Design and data collection

Active patient engagement and data collection for this paper started within two months after funding for the RECOVAC consortium was granted. In this phase the main parameters of the first RECOVAC studies were already selected, and data collection had started. This was caused by the rapid vaccine development and urgent roll-out of vaccination in high-risk groups due to the COVID-19 pandemic. Therefore, the RECOVAC consortium was established very fast and patient engagement started later.

To support data collection and to facilitate continuous learning on how patient engagement within RECOVAC was best implemented, Reflexive Monitoring in Action (RMA) was used. RMA is characterised by cycles of planning, action, observing, analysing, reflecting and adjusting activities [32]. In this project, RMA was implemented as a continuous feedback loop to jointly evaluate and reflect upon the patient engagement process, and when necessary, from an impact perspective, adjust and refine the engagement process.

To collect the qualitative data a participatory mixed methods approach [33] was used, including participant observations according to the definition of Schensul (“*the process of learning through exposure to or involvement in the day-to-day or routine activities of participants in the research setting“* [34]*)*, questionnaires with open-ended questions and interactive reflection sessions. The Patient Engagement Monitoring and Evaluation (PEME) framework was used to provide insight into the contribution of patient engagement in this specific project. The comprehensive PEME framework facilitates reflection and understanding on how patient engagement translates into impact and provides metrics to operationalize measurement of the different components of patient engagement. It consists of six main components, with 21 subcomponents that include a total of 87 metrics (i.e., measures) and 15 contextual factors [12]. Based on the criteria for selection of metrics, provided by the authors of the PEME framework, the framework was adapted for use in this study (see Table 1).

**Table 1:** Components and subcomponents of the PEME model [12] and application within this study

Component	Subcomponents and application within this study All included subcomponents are in bold in this column
**Objectives**	What is aimed to achieve with patient engagement in the RECOVAC consortium?
**Input**	Are the conditions for meaningful patient engagement in place? This component is divided into 4 subcomponents. ***Expectations*** refers to expectations all consortium participants have regarding patient engagement, but also to the needs, beliefs and priorities of everyone involved. Taking all this into account will enhance satisfaction, indirectly influencing learnings, changes and impacts. ***Preparations*** studies the accessibility (of preparatory materials) and preparedness of the patient engagement. It is believed that studies/consortia that are better prepared have better results. ***Resources*** as third subcomponent refers to materials, human and financial resources available to carry out the patient engagement. Dedicated time and funding are necessary to ensure meaningful and sustainable patient engagement. ***Representativeness of stakeholders***, assessing the expertise and diversity of stakeholders involved is the fourth subcomponent included. Carefully selecting patient partners is a critical step to obtain meaningful patient insights. The diversity of patient representatives is seen as a predictor for the diversity of learnings and recommendations.
**Activities and the process of patient engagement**	Monitoring activities and processes provide insight into the progress of implementation of patient engagement and what improvements can be made to enhance the implementation. Four subcomponents are important. ***Structure*** refers to the way patient engagement activities are organized: ‘what activities took place’ and ‘when’. ***Management*** refers to how patient engagement is facilitated. ***Interactions*** assesses the quality of the interactions between the stakeholders involved. ***Satisfaction*** includes the overall experience of those involved in the patient engagement initiative. Satisfaction is thought to be an important factor contributing to the willingness to collaborate and the overall value of patient engagement.
**Learnings and changes**	***Learnings*** refers to the insights and recommendations people deduce from the patient engagement activities. These learnings can be very diverse and stakeholder specific. Researchers may gain more insight in and better understand the experiences of patients’, while patients may learn about research processes. Metrics to measure learnings are challenging to define beforehand, as it is difficult to predict what you may learn from the patient engagement activities. Therefore, no metrics have been defined to measure learnings. ***Changes*** as a result of the patient engagement process refer to the actual adjustments made to the research and to changes on a more individual level, such as changing attitudes.
**Impacts**	If the acquired learnings and changes are put into practice, then long term impacts may be generated. Impact is studied using a wide variety of subcomponents, depending on the goals of patient engagement. The subcomponents that are included in this study are ***Research relevance***,*** study quality and efficiency***,*** reputation and trust*** and ***embedding of patient engagement***.
**Context**	When executing and assessing the contribution of patient engagement, it is essential to consider its ***context.*** Context has both an influence on the execution of the research, especially in this COVID research project, and on the impact the process and results will have in a later stage. ***Policy***,*** community*** and ***decision-making context*** are included in this study.

#### Participant observations

All e-mail conversations between policy officers, patient representatives and researchers were collected and qualitatively analysed. Moreover, during all meetings in the meeting structure (see results point 3), observational notes were made. The notes provided a reflection on the discussed points and the final decisions made (if any) and the process of patient engagement. The analysis of the notes was done together with the qualitative analysis of the other observational data and followed the same structure.

#### Questionnaires with open ended questions

To provide insight in the perspectives of researchers and patient representatives on the objectives, expectations, experiences, changes and impact of patient engagement, one of the patient representatives (JPMV) and the policy officers (WSK, RWL) of the NVN developed a questionnaire consisting of 10 open-ended questions based on the PEME model (see Annex I for full questionnaire). Respondents were asked to fill in the questionnaire twice - at the beginning of the patient engagement activities, and three months after the start of the activities - to provide insight in changes in their perspectives regarding the contribution of patient engagement over time. Only respondents who filled in the first questionnaire were asked to respond to the second questionnaire.

#### Interactive reflection sessions

To validate and jointly reflect on the analysis of the observations and questionnaires, interactive reflection sessions were organised. These sessions provided additional insights in underlying opinions, arguments and experiences regarding the patient engagement process. Topic guides (see Annex II) were drafted based on the PEME model, and the analysed data from the questionnaires and the observations. Preliminary results were shared with participants during the session via a short summary presentation and the topic guide structured the discussion that followed. Sessions were either semi-structured focus groups or individual interviews, depending on the preferences of the participants. First, sessions with researchers were organized (one focus group, three individual interviews), followed by one focus group with patient representatives.

### Participants and recruitment

Participants were recruited via email or personal contact. Participation was only possible for members (both researchers and patient representatives) of the RECOVAC consortium. Recruitment for the first-round questionnaire started in the second quarter of 2021 and all members of the consortium that were involved in patient engagement were invited to participate. For the reflection sessions, recruitment started when enough data on all major components was collected to reflect on the patient engagement activities, in the second and third quarter of 2022. All patient representatives and a representative selection of researchers, based on their role within the consortium, were included in the reflection sessions.

### Data analysis

The qualitative data from the participant observations and the questionnaires were analysed based on the components of the PEME framework. One of the patient representatives (JPMV) analysed the data together with the two policy officers of the NVN (WSK, RWL) using a thematic approach [35] in which data was coded and outcomes were categorized according to the components and subcomponents of the PEME framework. Both the patient representative and the two policy officers have extensive experience in performing qualitative analysis, based on their master’s degree and previous research experience. The results were shared and discussed within this team and validated and complemented in the reflection sessions. The additional data collected in the reflection sessions were analysed in the same thematic way.

### Quality control

The set-up of this process evaluation followed the sequence of planning, action, observing, analysing and reflecting in which multiple participants were involved. The questionnaires and topic guides for the reflection sessions were designed by multiple researchers (JPMV, WSK and RWL) in an iterative way. Data analysis was done by multiple researchers (JPMV, WSK and RWL) in such a way that data was always analysed by two researchers independently of each other, mutually discussed in the team of three and finally validated in the reflection sessions.

## Results

### Descriptive results

In the first round of questionnaires, 16 researchers received the questionnaire of which seven responded and of which four completed the follow-up questionnaire. All patient representatives, in both the first and second round, completed the questionnaire. For the reflection sessions with researchers a selection of 14 researchers was invited of which eight participated. Five researchers participated in a focus group and three researchers had an individual interview. For the reflection session with patient representatives all five representatives were invited and participated in the session.

### Patient engagement assessment results

The results section is built along the six main components, including the selected sub-components, of the PEME framework, following the structure of Table 1.

### 1. Objectives

Although the exact aim of patient engagement was not well-defined prior to the study, it was clear that patient engagement would be an important component within the consortium. In the research proposal it was stated that patient engagement would be an integral part via a patient advisory board, meaning that patients would be engaged in “all stages of the research project, including but not limited to, development of the protocol, realization of the study, reporting, implementation and communication of results”.

### 2.Input

#### Expectations

Expectations, prior to the start of the studies in the consortium, differed between patient representatives and researchers. Researchers expected that involving patients would contribute somehow, but because of limited experience with patient engagement, they could not explicate what that contribution would entail. They were unsure about the exact role of patient representatives: should patient representatives only speak from daily experience and their feelings and emotions, or could they also participate in scientific discussions? Should they only be involved in the communication aspect of the studies, or could they also be involved in decisions about the study design? As opposed to the researchers, patient representatives had clear expectations on their contribution with both knowledge from and experience with daily practice and with their views on the scientific part of the studies. These clear expectations were based on their extensive experience with patient engagement in research. However, patient representatives were sceptical whether they would be able to contribute in a meaningful manner and if they would manage to become a serious partner within the consortium.

#### Preparations

The preparations for patient engagement were not optimal according to both the researchers and the patient representatives; a direct result of the exceptionally fast pace of the activities in the consortium caused by the COVID-19 pandemic, leading to a rushed proposal and start-up phase. Both researchers and patient representatives indicated that – at the start of the activities – it was not clear who was responsible for defining objectives and for deciding in which specific processes engagement of patients was necessary and useful. Consequently, researchers did not always know what they could ask patient representatives and patient representatives did not feel well prepared and involved. Therefore, it took longer to establish a clear working relationship and to build mutual trust, with patient representatives indicating that it felt as if they first had to earn their position within the team, and that it took them a while to feel that they were a serious partner in the consortium.

#### Resources

In the consortium there was budget made available for patient engagement, which was to be organized, facilitated, and evaluated by the NVN. This budget was sufficient to cover part of the time invested by the NVN; patient representatives participated on a voluntary basis.

The time required to ensure a satisfactory contribution from the patient representatives resulted in challenges for both researchers and patient representatives. Researchers indicated that they had to find time to prioritize patient engagement, as it was an extra task that was added to their already heavy workload. Patient representatives found that the time they had to collect input (patient experiences, questions, opinions etc.) from the group they represented and for discussions among themselves, before having to report back to the researchers, was too limited. This was due to the tight deadlines within the research project itself.

#### Representativeness of stakeholders

The group of patient representatives was representative for a broad range of type of kidney patients. On many issues the patient representatives collected broader input from the patient community to ensure a broad range of perspectives, for example via nieren.nl, via the questions that came in at the NVN and via several reliable Facebook groups. The patient representatives perceived COVID-19 as a serious threat and followed strict protective measures. However, not all patients felt and acted the same. The collected information made it possible for the patient representatives to also include other voices regarding COVID-19 threat in each advice.

The group of researchers involved in the project was diverse. It consisted of clinical and non-clinical researchers at different stages of their career from the departments of Nephrology, Microbiology and Immunology, with different levels of patient engagement experience. Some researchers indicated that they had much experience, e.g., the project coordinators and their teams, some researchers indicated that they had a bit of experience and some indicated that they had no experience with patient engagement prior to the project.

According to both researchers and patient representatives, it is important for patient representatives to have sufficient experience with and knowledge about scientific research in general and patient engagement specifically to be able to get quickly involved in a meaningful way in such a complex high paced project. They indicated that extra training and teaching moments to reach the level of knowledge and skills needed to participate adequately in this project, would have been needed when less experienced patient representatives would have been included.

### 3. Activities and process

#### Structure

Designing the structure of the patient engagement activities within this project was a collaborative process between the researchers, the NVN and the patient representatives. It was jointly decided that the patient representatives had a role in each step. This ranged from joining (almost) all consortium meetings, co-creating communication materials for patients, collectively interpreting data and making joint decisions including co-authorship on several scientific publications. Interaction between patient representatives and researchers was not limited to the meetings, but also took place via email and, if necessary, via separate dedicated meetings.

Within the meeting structure, three different kinds of meetings had an influence on the patient engagement process. First, the meetings of the Steering Committee of the consortium, in which one patient representative was present on behalf of all patient representatives. This patient representative was also contact point for all questions. Participation in these meetings was necessary to better understand the context of the studies, to provide input, to participate in discussions and decision making and to answer questions on patients’ needs and perspectives. It increased visibility and understanding among researchers and patients felt sufficiently involved and informed to participate in a meaningful way. Secondly, the meetings of the patient representatives facilitated by the policy officers of the NVN. Main goals of these meetings were to discuss and formulate feedback and answers on questions from the researchers and to continuously reflect on the role of patient engagement within the consortium via RMA. Thirdly, the meetings between the patient representatives, the policy officers and the two project coordinators which started later in the project in response of the needs of the patient representatives and the research team. These meetings were seen as crucial by the patient representatives and the project coordinators as these ensured in-depth discussions on certain topics, creating mutual understanding and increasing the effectiveness of the partnership in the consortium. In these meetings RMA was also applied, resulting in continuous reflection on the process and swift adaptation when needed.

#### Management and interactions

All parties within the consortium recognized that there was willingness, time and space to implement patient engagement, within as well as outside meetings.

During the consortium meetings, patient input was always an item on the agenda. In addition, there was room to introduce discussion points, questions or concerns and to respond to questions from the researchers. When a question could not be answered immediately, patient representatives had time outside of the meetings to discuss and to formulate a broad patient perspective, making it easier to share a thorough broad-based standpoint or wish with the researchers.

All parties involved regarded the interactions between researchers and patient representatives as very positive; there was mutual respect. The researchers were impressed by the capabilities of the patient representatives, their ability to participate in the high pace of the consortium and the knowledge level they had.

#### Satisfaction

The researchers highly valued the feedback, knowledge, attitude, and commitment of the patient representatives. They felt that patient representatives had an influence on many aspects of the research. From the start, patient representatives felt taken seriously and noticed their input was being valued and used, resulting in a feeling of appreciation – although this fluctuated during the project. Patient representatives were not always satisfied with the impact of their input and feedback, which resulted in feelings of frustration at some points in time. However, later in the project, during the 6-weekly meetings between the patient representatives and the project coordinators context and choices were explained and elaborately discussed, allowing for a turn-around of these unsatisfied feelings. Understanding of the context, having insight in the decision-making process and seeing the same frustrations among researchers via these meetings, turned out to be crucial for patient representatives’ satisfaction.

### 4. Learnings and changes

#### Learnings

Researchers indicated that the active involvement of patient representatives in meetings helped them to gain insights into the experiences of patients and the influence their research activities would have on patient’s daily lives. Especially early career researchers and researchers who have limited patient contact said, for example, that they underestimated how emotionally charged the research and its outcomes would be for patients, increasing their sense of urgency for their research. Furthermore, knowledge among researchers about patient engagement increased. They shared that they learned, for example, under which conditions patients can participate, what difficulties could be encountered and how to deal with them.

Patient representatives indicated that they learned more about participating in a consortium and that they better understand how their feedback is handled considering all other interests within the consortium and its context. They found that they gained new insights and grew into their role, which, in their opinion, improved the collaboration over time. They were already experienced in patient engagement, but due to the intensive nature of the engagement process in this consortium, they became more efficient and effective in their contributions. They learned that an important part of their role was to help bridge the gap between theory/science and daily life with a kidney disease in times of COVID-19. An example of such a gap was the contradiction in needs on communication of results: for patients, dissemination of preliminary results is of major importance while in scientific practice this is usually not done. This resulted in new ways of sharing results with patients, for example through recurring webinars before results were scientifically published.

#### Changes

The engagement of patient representatives in the research did not change the main objectives, since these objectives already related to the needs of patients. Adjustments were only made to specific elements related to the set up and execution of the studies. The main changes were: (1) adding a cohort study which aimed to assess efficacy of COVID-19 vaccination in patients with either severely impaired kidney function, on dialysis or living with a kidney transplant in comparison to non-vaccinated control groups to the list of studies in the consortium; [28] (2) removing one of the suggested study arms in a randomized controlled trial that was designed to investigate whether immunogenicity of COVID-19 vaccination could be increased, because the burden of that arm for patients was deemed too high by the patient representatives; [26] (3) adding a questionnaire to gain insight in the change in self-reported adherence to government implemented behavioral measures to prevent COVID-19 after vaccination and to compare adherence between groups with different antibody responses after vaccination; [25, 36] and (4) sending all participants their individual antibody results. Moreover, all communication materials for both participants and the wider (general) audience were reviewed by the patient representatives resulting in easier to understand letters, more informational webinars to share results and discuss questions and fears of patients than originally planned and in adaptations in the project website.

The involvement of patient representatives within the consortium also changed clinical practice of some participating nephrologists. Some nephrologists, for example, indicated that the knowledge they gained from the patient representatives influenced the conversations with their own patients. They became more aware how COVID-19 impacted daily life of patients and of the patient’s needs regarding information and therefore the importance of reliable and understandable sources of information.

### 5. Impact

Patient engagement within the RECOVAC consortium had impact on the conduct and outcomes of the research activities in the consortium and may have impact on future research and patient engagement activities in general. According to both the researchers and the patient representatives, the input of the patient representatives improved the current research as the results were more meaningful and better aligned to the needs and daily lives of patients. Also, by being part of the consortium, patient representatives had direct information and indirect influence on decisions made by the National Institute for Public Health and the Environment (RIVM) on preventive measures and treatment against COVID-19, like the vaccination schedule and the availability of medication. Moreover, both researchers and patient representatives indicated that they disseminated their learnings beyond the scope of the project activities. As a result, the NVN noticed an increased and timelier number of questions and requests on patient engagement coming in from researchers involved in the RECOVAC consortium. Also, the policy officers from the NVN indicated that the insights and experiences generated within this patient engagement process, such as the importance of a good preparation phase in which expectations and objectives are clarified, are already applied to new patient engagement activities guided by the NVN.

### 6. Context

The societal impact of the COVID-19 pandemic was enormous, making the community context extremely important and influential during this project. COVID-19 was a new disease with major consequences for all aspects of life, especially for high-risk groups such as kidney patients. Because of these major consequences, all information was heavily scrutinized and highly decisive for how this patient group would live their life. Patients expressed their questions and frustrations through many different channels and the patient representatives emphasized these worries and fears to the researchers so that the researchers felt they could not ignore this and understood the urgency and need for good and clear communication as described above.

This consortium addressed a complex problem. The decisions that were made within this consortium did not only involve considerations of the researchers, patient representatives and the involvement of the local and regional stakeholders, but also a great number of other stakeholders. Some decisions were made on national and European level, for example which vaccines or medicines would become available and when. Because of this high level of stakeholder influence, maximum impact of patient engagement was not always possible, or the requested changes could not be made because they were not within the sphere of influence of the consortium. Patient representatives indicated that this was frustrating at times and researchers expressed similar difficulties. However, it did offer patient representatives the opportunity to get information straight from the source which made it possible for them to receive and share information fast within the larger group of kidney patients.

According to the researchers, the Dutch policy context also played an important role in the possibilities of the research project and beyond. The COVID-19 vaccination policy in The Netherlands was coordinated by the RIVM, commissioned by the Dutch Ministry of Health. A working group, including medical specialists from all relevant fields, was formed to discuss COVID-19 vaccination in high-risk and immunocompromised patients. This working group, which included two members of the RECOVAC consortium, formulated advice on vaccination efficacy and frequency for groups at risk for severe COVID-19 outcomes. Taking input from this working group into account, decisions on (repeated) COVID-19 vaccination were made by the Minister of Health. This decision process was not always clear and transparent, but decisions made did have a high impact on how and when certain studies of the RECOVAC project could be executed.

## Discussion

The aim of this study was to provide insight in the contribution of patient engagement in the specific context of the RECOVAC (Renal Patients COVID-19 VACcination) consortium and provide practice-based action perspective for future patient engagement trajectories. Based on the results found per component, we can say that in this study the objectives were not well defined prior to the start of the patient engagement trajectory, but both researchers and patient representatives agreed on the high importance of patient engagement in the project. There are some points of improvement with regard to the input component, for example it is needed to have more budget, better alignment on expectations and more time to establish working relations. The activities and process component shows that co-creation has taken place in establishing the working process, guided by RMA, which made it possible for patient engagement to be fully integrated in this study. Satisfaction fluctuated, but that was always resolved by staying in good contact with each other and by transparent communication. We have seen many learnings and changes in the project. Research activities have been adapted, e.g., adding a cohort study, removing a suggested study arm, adding a questionnaire on behavioural measures and sending participants their individual antibody results. Beyond the project learnings have influenced the clinical practice of involved clinicians as well as the way in which the NVN provides guidance to patient engagement in other research projects. The context component was also important, because it influenced the possibilities of the researchers to implement wishes from patient representatives.

We have found that by using the PEME framework, we could provide insight in the process of patient engagement in a research project and that the course of a research project may change based on the input given by patient representatives. Due to these changes, there was immediate impact on the daily lives of the patients in some areas, for example knowing their individual antibody results [36]. We have also found that making use of Reflexive Monitoring in Action helps to improve the patient engagement process while the project is running. Due to the adaptations made, the patient engagement activities were tailored to the specific needs of the project and of the patient representatives. This resulted in an adjusted project course and influence on the subsequent results.

### Recommendations: practice based action perspectives

Due to the structured and thorough method of providing insight in the contribution of the patient engagement process, several concrete practice-based action perspectives have been formulated to help reduce functional task uncertainty, which do need contextualisation to fit the specific project they will be applied in.

#### Invest in building a relationship, by engaging patients as early as possible and making them an integral part of the team

Our study shows that when you start active patient engagement after the research proposal has already been written, it takes more effort to get the patient engagement process up and running in such a way that it becomes truly a collaborative effort in which all parties feel comfortable. This finding is also supported by several other studies [13, 20, 37, 38].

#### Jointly formulate clear patient engagement objectives and discuss expectations and structure of the collaboration before starting

Starting off with clear objectives, expectations and structure, collaboratively formulated, is a prerequisite for a well-structured patient engagement process. Multiple studies show that both researchers and patient representatives feel the same need [37, 39]. The PEME framework provides guidance for this process [12].

#### Use the RMA approach to evaluate and adjust the patient engagement process continuously throughout the project

Reflecting on the patient engagement process, by using the RMA approach, strengthened mutual understanding, resolved outstanding issues and improved patient engagement activities throughout the project. This is a finding that is supported by the experience of cancer patient representatives in the CanIMPACT study [38] and in the review of Harrison [13], who also adds that evaluation contributes to patient representatives being active partners and keeping them informed in the study.

#### Be transparent about the effects of the efforts of the patient representatives, by explaining what is done with the provided input and explaining why some advice cannot be incorporated or only in an adapted form

This is important for patient representatives to stay motivated and to create a learning effect. The patient representatives in the CanIMPACT study indicated that they had similar doubts about their added value as the patient representatives in this study and the same need for transparency [38].

#### Make the effort needed for patient engagement to become an explicit topic of discussion, both for researchers and patients

Patient engagement takes considerable effort and time from both researchers and patient representatives, given their specific circumstances (e.g., high workload for researchers, limited energy for patient representatives impacting the workload that can be handled). Therefore, the organization of the process needs to be explicitly discussed in order to make agreements on how to execute it most efficiently for all parties involved [37, 39].

#### Include trained and/or experienced patient representatives to be able to achieve high quality patient engagement

The fact that patient representatives had prior experience with patient engagement and with research in general showed to be of vital importance. If patient representatives have no or little prior experience, it is important to provide training. This is reinforced by findings from Kirwan et al. [20] Forsythe et al [39], Carroll et al. [40] and Heijerman-Holgrefe et al [41].

#### Discuss with all stakeholders involved in the patient engagement process which budget is needed to adequately facilitate patient engagement, what that budget is meant to be spent on and incorporate that in the budget for the project

Our study found that to be able to facilitate high quality patient engagement it is important to have sufficient budget available, for example to be able to include efforts from a patient association. Several other studies have also shown that fair reimbursement for patient associations, and for patient representatives as well, is a key component when setting up patient engagement in health research [13, 20, 37, 42]. There are models and guidelines available that provide insight in fair compensation for patient representatives in different situations [43, 44]. Context always needs to be taken into account when reimbursement is discussed.

### Strengths and limitations

A strength of this study is the systematic assessment of patient engagement in a fast paced, highly complex project that has direct impact on the daily lives of patients. An added value to this systematic assessment is that it was performed by a unique group of people. All stakeholders involved in the RECOVAC activities were actively involved in the assessment of the patient engagement process, both as participants in the study and as authors of the publication, ensuring widely supported recommendations.

This broad collaboration of stakeholders in the assessment also resulted in direct impact of the learnings beyond the scope of the RECOVAC project. For example, the NVN is highly valued in the field of nephrology research, which gives weight to the results of this study and also has implications for the direct transferability and implementation of the lessons learned from this assessment in other research projects. The NVN is already applying learnings from this assessment to other research projects (e.g., training of patient representatives). This will ensure a long-term effect of lessons learned in future patient engagement activities in health research.

A limitation of this study is the stage in which the patient representatives joined the research project. In an ideal situation you would want to be involved from the conception of the idea, before the grant application for the funding is being written. In this case patient representatives joined right after the grant was awarded. This had to do with the very short deadline for submitting the research proposal, as that was at the hight of the COVID-19 pandemic and speed was of the essence. A second limitation is the low response rate in the second round of questionnaires that was sent to the researchers, which might have been caused by the high workload given the extreme situation of the pandemic. Only four researchers responded. However, the impact of this low response rate on the results seems minimal as the results from the researchers that did fill in the questionnaires in round two were very similar to the answers given by all researchers that participated in round one. More importantly, the results were confirmed by the observations done during the patient engagement process and the consecutive interactive reflection sessions.

## Conclusion

This study provides insight in the contribution of patient engagement within the RECOVAC consortium. Because of its unique context, this patient engagement process has been a special journey with direct influence on patients’ lives, the research plans and outcomes of the project, but also on the daily practice of the researchers involved. The researchers gave space and were receptive towards the input of patient representatives. And despite the high pace of the project, patient representatives were willing and able to invest a lot of time and energy into the project. This has resulted in a patient engagement process that was able to provide insight in what the added value of patient engagement in health research can be and evidence based concrete action-based perspectives. The lessons learned in this project can help to prepare and implement patient engagement in future research projects.

## Electronic supplementary material

Below is the link to the electronic supplementary material.

Supplementary Material 1

Supplementary Material 2

## Data Availability

The data generated and analysed during the current study are not publicly available due to privacy issues.

## Declarations

### Author information

The author team consists of authors with a mix of different backgrounds. The main author (JPMV) is a patient representative with an extensive background in patient engagement in research. She is also a PhD student at the Global Health Unit of the department of Health Sciences at the University Medical Center Groningen. This article will be part of her PhD thesis. WSK, MMHP and RdWL are involved on behalf of the Dutch Kidney Patients Association (NVN) and RTG, ALM and JSFS are clinicians and researchers and involved on behalf of the RECOVAC consortium. DEMCJ, CB and JdZ are the supervisory team of the PhD trajectory of JPMV.

## Abbreviations

RECOVAC: Renal Patients COVID-19 VACcination
PEME framework: Patient Engagement Monitoring and Evaluation framework
RMA approach: Reflexive Monitoring in Action approach
NVN: Dutch Kidney Patients Association
RIVM: National Institute for Public Health and the Environment
