# Supplementary material for: Patient engagement as a collaborative process in a large Dutch COVID-19 vaccination study (RECOVAC) – insight into the contribution of patient engagement and learnings for the future
Source: Res Involv Engagem. 2024 Sep 13;10:96. doi: 10.1186/s40900-024-00622-x (PMC11395945; doi:10.1186/s40900-024-00622-x)
Supplement: Supplementary file 1 — Supplementary Material 1 [file 40900_2024_622_MOESM1_ESM.docx]

**S1 Annex 1 – questionnaire**

**FOR RESARCHERS**

**Questionnaire 'Patient engagement within the RECOVAC consortium**

**This questionnaire consists of 10 open-ended questions. You can use the text boxes for the answers. You can adjust the size of the text boxes yourself if you need more writing space.**

**Part 1 goals and expectations (only asked in round 1)**

**Question 1**. Why did you decide to involve patients in the project? With what purpose? What did you hope to achieve with engaging patient representatives?

**Question 2**. What were your expectations regarding the added value of patient engagement? What did you think it would add to the consortium?

**Question 3**. What were your expectations regarding the process, the form of patient engagement? How do you think the process will go? For example, what kind of positive and negative expectations did you have?

**Question 4.** To what extent did you feel prepared to involve patients in the process?

- What was this feeling based on? How has this affected the process?
- What preparations have been made for patient engagement?
- Looking back: what would you have done differently? Which preparations would have been better/necessary?

**Part 2 experiences and effects**

**Question 5.** To what extent are you satisfied with patient engagement? Think of the process, but also the result of patient engagement. How does this relate to the expectations you had beforehand?

**Question 6.** What has changed recently with regard to patient engagement within the project? Consider, for example, how patient engagement is designed and how it is ensured that patients can share their experience and vision.

**Question 7.** What input from patient representatives was a real eye-opener for you and/or the project?

**Question 8.** Which actions and adjustments in the project have been made because of input from the patient representatives?

**Question 9**. How has your attitude towards patient engagement changed through experiences in this project? To what extent have you seen the attitude towards patient engagement of others change within the working group?

**Question 10.** What have you learned about patient engagement in the past period?

**FOR PATIENT REPRESENTATIVES**

**Questionnaire 'Patient engagement within the RECOVAC consortium**

**This questionnaire consists of 9 open-ended questions. You can use the text boxes for the answers. You can adjust the size of the text boxes yourself if you need more writing space.**

**Part 1 goals and expectations (only asked in round 1)**

**Question 1.** Why did you join the consortium as a patient representative? With what purpose? What did you hope to achieve?

**Question 2.** What were your expectations regarding the added value of patient engagement? What did you think it would add to the consortium?

**Question 3.** What were your expectations regarding the process, the form of patient engagement? How do you think the process will go? For example, what kind of positive and negative expectations did you have?

**Question 4.** To what extent did you feel prepared to be involved as patient representative?

- What was this feeling based on? How has this affected the process?
- What preparations have been made for patient engagement?
- Looking back: what would you have done differently? Which preparations would have been better/necessary?

**Part 2 experiences and effects**

**Question 5.** To what extent are you satisfied with patient engagement? Think of the process, but also the result of patient engagement. How does this relate to the expectations you had beforehand?

**Question 6.** What has changed recently with regard to patient engagement within the project? Consider, for example, how patient engagement is designed and how it is ensured that patients can share their experience and vision.

**Question 7.** To what extent do you feel involved in the project?

- Is there enough room for interaction and input?
- Do you feel taken seriously?

**Question 8.** Which actions and adjustments in the project have been made because of input from the patient representatives?

**Question 9.** What have you learned about patient engagement in the past period?

**S2 Annex II – topic guides interactive reflection sessions**

**FOR RESARCHERS**

**Questions about 'input'**

It is important to formulate clear and specific objectives in advance with regard to patient engagement. Based on the grant proposal and the completed questionnaires, it seems that this has not been the case for the current project.

- Do you recognize this? To what extent did you miss the fact that no concrete objectives had been formulated?
- What is needed to improve this (formulation of clear objectives beforehand) for future patient engagement in research projects?
  - To what extent does previous experience with patient engagement also play a role in this?
  - To what extent does it have to do with the context in which the research project takes place?

Proper preparation and facilitation of patient engagement is also an important part of a successful engagement process. A project such as this research project is unique in its set-up and, partly due to the context in which it takes place, moves very quickly. The lead times are short, ad hoc action must be taken regularly, which means that keeping a good helicopter view can be a challenge. This is a situation that can demand a lot from a person, both for the researchers and for the patient representatives in the research group. As a result, optimal patient engagement is not always possible.

- What could have been or was done to better prepare and facilitate patient engagement, despite the complex context of the project, in order to optimize patient input without asking too much of the patient representatives?
  - To what extent is the facilitation of patient engagement different from what you expected beforehand?

Another important aspect of patient engagement is the composition and representativeness of the group of patient representatives.

- What do you, as researchers, expect in terms of representativeness (with regard to the entire target group) of patient representative? Do they represent the entire target group in your eyes or is it just one of the target group's opinions that you take into account when conducting research?
- What level of subject matter expertise do you expect from patient representatives? Both in terms of the specific research topic and in terms of knowledge on how to do research in general.
  - Patient engagement is now often on the agenda as “communication from patient representatives”, is that also the expectation with regard to patient engagement?
- Do you also take experiences from your own daily practice into account when judging the representativeness of the patient representatives involved in the patient engagement process? If so, how do they relate to each other?

**Questions about 'activities and process'**

A good structure of patient engagement is important to generate impact. In this project, the agreements about the structure and perhaps expectations regarding the structure were not very clear. This relates to the role that the patient representatives can play within the research, both very practically in the meetings and, for example, when providing feedback on documents or providing input on communication tools.

- To what extent is the way in which patient engagement is organized now in line with what it should yield within the research?

The completed questionnaires show that researchers (and also patient representatives) are satisfied with the interaction between patient representatives and researchers. This is also reflected in the meetings that were held between the patient representatives, the policy officers and the two project coordinators which started later in the project.

- What are the reasons for this satisfaction among the researchers?
  - What specific contribution do patient representatives make that causes this satisfaction?
- In retrospect, what should have been done differently to get more out of patient engagement?

**Questions about 'learnings and changes'**

Due to the input of patient representatives, the project has changed in all kinds of areas.

- What would you have overlooked if patient representatives hadn't brought it in?
- To what extent are the changes made to the study a compromise or do you really support the change? A clear example of this is the adjustment of the arms in one of the studies and the sending of the individual antibody results.
- What do you think of the quality of the contribution made? Is it better or worse than you expected?

In addition to changes to the project, we also hope that everyone involved has learned something.

- What have you learned from or via patient engagement in this project?
  - About the added value of patient engagement?
  - About how you execute patient engagement?
  - New insights that you have obtained through input from patient representatives?
  - Other, broader, learning points?

**Questions about 'impact'**

If the lessons learned are applied in practice, long-term impact/changes can occur.

- What do you think is the long-term impact of patient engagement?
  - What impact has this patient engagement process within RECOVAC had on you personally?
  - And what do you think is the impact on the renal field? What are the long-term effects on, for example, research in the renal field?

**Questions about 'context'**

The context has an important influence on the course and outcomes of patient engagement. The context of the RECOVAC research is unique because the research was so intertwined with everyday life. We are very curious about the influence of this unique context on patient engagement.

- How did external influences determine patient engagement in this project? Influence on: Satisfaction, added value, time and resources, possibilities, impact, etc.
- National policy
- Involvement of many medical centers/partners
- Media

In the discussions, the difference between scientific based choices and outcomes and their application in daily practice often emerges. Given the urgent context (acute risk of infection + associated consequences), patient representatives were very interested in what the results mean for daily practice and how they can be applied. This was not always in line with the wishes and views of the researchers.

- How did you experience this friction, caused by the different perspectives? (Science vs. translation to direct daily practice)

**FOR PATIENT REPRESENTATIVES**

**Questions about 'input'**

It is important to formulate clear and specific objectives in advance with regard to patient engagement. Based on the grant proposal and the completed questionnaires, it seems that this has not been the case for the current project.

- Do you recognize this? To what extent did you miss the fact that no concrete objectives had been formulated?
- What is needed to improve this (formulation of clear objectives beforehand) for future patient engagement in research projects?
  - To what extent does previous experience with patient engagement also play a role in this?
  - To what extent does it have to do with the context in which the research project takes place?

Proper preparation and facilitation of patient engagement is also an important part of a successful engagement process. A project such as this research project is unique in its set-up and, partly due to the context in which it takes place, moves very quickly. The lead times are short, ad hoc action must be taken regularly, which means that keeping a good helicopter view can be a challenge. This is a situation that can demand a lot from a person, both for the researchers and for the patient representatives in the research group. As a result, optimal patient engagement is not always possible.

- What could have been or was done to better prepare and facilitate patient engagement, despite the complex context of the project, in order to optimize patient input without asking too much of the patient representatives?

Another important aspect of patient engagement is the composition and representativeness of the group of patient representatives.

- What do you, as patient representative, expect in terms of representativeness (with regard to the entire target group) of patient representatives? Have you been able to represent the broad group of kidney patients?

**Questions about 'activities and process'**

A good structure of patient engagement is important to generate impact. In this project, the agreements about the structure and perhaps expectations regarding the structure were not very clear. This relates to the role that the patient representatives can play within the research, both very practically in the meetings and, for example, when providing feedback on documents or providing input on communication tools.

- To what extent is the way in which patient engagement is organized now in line with what it should yield within the research?

The completed questionnaires show that researchers and patient representatives are satisfied with the interaction between patient representatives and researchers. This is also reflected in the meetings that were held between the patient representatives, the policy officers and the two project coordinators which started later in the project.

- What are the reasons for this satisfaction among the patient representatives?
- In retrospect, what should have been done differently to get more out of patient engagement?

**Questions about 'learnings and changes'**

Due to the input of patient representatives, the project has changed in all kinds of areas.

- What do you think has changed based on the input you brought in as patient representatives?
- What are you most proud of with regard to the patient engagement activities you have been involved in?

In addition to changes to the project, we also hope that everyone involved has learned something.

- What have you learned from or via patient engagement in this project?
  - About the added value of patient engagement?
  - About how you execute patient engagement?
  - New insights that you have obtained through the patient engagement activities?
  - Other, broader, learning points?

**Questions about 'context'**

In the discussions, the difference between scientific based choices and outcomes and their application in daily practice often emerges. Given the urgent context (acute risk of infection + associated consequences), patient representatives were very interested in what the results mean for daily practice and how they can be applied. This was not always in line with the wishes and views of the researchers.

- How did you experience this friction, caused by the different perspectives? (Science vs. translation to direct daily practice)
